# Phenothiazines enhance antibacterial activity of macrophage by inducing ROS and autophagy

**DOI:** 10.3389/fimmu.2025.1712724

**Published:** 2025-11-28

**Authors:** Lihao Qiu, Wen Chen, Jianfeng Wang, Xuming Deng, Hongtao Liu, Jiazhang Qiu

**Affiliations:** State Key Laboratory for Diagnosis and Treatment of Severe Zoonotic Infectious Diseases, Key Laboratory for Zoonosis Research of the Ministry of Education, College of Veterinary Medicine, Jilin University, Changchun, China

**Keywords:** phenothiazines, intracellular bacteria, HDTs, ROS, autophagy

## Abstract

Bacterial infections pose a major global public health threat. While antibiotics have historically served as the primary treatment, the rapid rise of antibiotic resistance has led to an urgent need for new therapeutic strategies. Host-directed therapies (HDTs), which activate defense mechanisms of host cells, are emerging as a promising alternative. Host-acting compounds (HACs) have no direct effect on bacteria and therefore do not induce drug resistance or alter intestinal microbiota composition. In the present study, we demonstrated that phenothiazines significantly enhance the antibacterial capacity of macrophages. In macrophages treated with phenothiazines, we observed a significant increase in lysosomal activity, induction of autophagy, and accumulation of reactive oxygen species (ROS). Importantly, co-treatment with autophagy inhibitors or ROS scavengers markedly diminished the antibacterial effects of phenothiazines. Furthermore, perphenazine (PHZ) effectively reduced organ lesions and inflammation associated with *S.* Typhimurium infections *in vivo*. Our results demonstrated that phenothiazines are lead compounds for antibacterial agents via HDTs.

## Introduction

Bacterial infections cause more than ten million deaths annually worldwide and represent a significant global public health threat (1, 2). Antibiotic therapy remains the primary approach for managing bacterial infections (3). However, the increasing prevalence of antimicrobial resistance (AMR) has raised serious concerns, and drug-resistant bacterial infections are projected to become the leading cause of mortality by 2050 (4–6). Furthermore, intracellular pathogens such as *Salmonella enterica* serovar Typhimurium (*S.* Typhimurium), *Shigella flexneri* (*S. flexneri*), *Staphylococcus aureus* (*S. aureus*) and *Listeria monocytogenes* (*L. monocytogenes*) reside within host cells, which presents a challenge for conventional antibiotics to effectively target them (7, 8). As a result, these intracellular pathogens are difficult to eradicate, complicating the treatment of associated infectious diseases (9, 10). Therefore, there is an urgent need for innovative therapeutic strategies and pharmaceutical interventions to address bacterial infections more effectively.

Macrophages are a type of innate immune cell that are the first line of antibacterial defense (11). After being engulfed by macrophages, bacteria are destroyed by the innate immune mechanisms such as autophagy, inflammatory responses and ROS (12–14). However, several intracellular bacterial pathogens have evolved numerous strategies to circumvent the host immune response during prolonged host-pathogen interactions (15). The *S. flexneri* effector VirA inactivates Rab1 GTPase through its GTPase-activating protein (GAP) activity, leading to inhibited autophagosome formation and suppressed autophagy (16, 17). The effector protein NleE from *Enteropathogenic Escherichia coli* specifically modifies a cysteine in the zinc finger domain of TAB2/3 through its methyltransferase activity, leading to a loss of the ability to bind ubiquitin chains and the inhibition of the activation of the NF-κb signaling pathway (18). Moreover, several intracellular bacteria, such as *Legionella pneumophila* and *Salmonella enterica*, possess the capacity to induce metabolic reprogramming of macrophages in order to facilitate their intracellular proliferation (19, 20). All these factors have led to heightened challenges in the eradication of intracellular bacteria. In the face of this medical dilemma, host-directed therapies (HDTs) based on HACs provide more options for combating bacterial infections (21, 22). Previous studies have shown that phenothiazines, a class of antipsychotic medication, have the ability to inhibit bacterial intracellular replication (23–25). However, the molecular mechanism by which phenothiazines inhibit the intracellular replication of bacteria has not been elucidated.

Here, we revealed that phenothiazines enhance the antibacterial activity of macrophages. Phenothiazines modulated glucose metabolism and induced the accumulation of ROS in macrophages, which promotes the activation of autophagy and lysosomal activity, thereby enhancing the antibacterial ability of macrophages. In addition, PHZ exhibited therapeutic efficacy against bacterial infections in animal models. This study provided evidence that phenothiazines may serve as lead compounds under HDTs for the management of bacterial infections.

## Materials and methods

### Bacterial strains and gentamicin protection assay

*S.* Typhimurium SL1344, *S. flexneri* 2a 2457T, *S. aureus* ATCC29213 and *L. monocytogenes* ATCC19115 were utilized in the gentamicin protection assay. RAW 264.7 cells were infected with bacteria at a multiplicity of infection (MOI) of 10. During the gentamicin protection assay, macrophages were infected for 30 minutes. Subsequently, a fresh culture medium containing 100 µg/mL of gentamicin was added to eliminate extracellular bacteria for 1 h, followed by treatment with fresh culture medium containing 20 µg/mL of gentamicin for 8 h. During this process, various concentrations of phenothiazines (promethazine (APExBIO, #B4784), trifluoperazine (APExBIO, #B1397), chlorpromazine (APExBIO, #B1480), mesoridazine (APExBIO, #B8701) and perphenazine (APExBIO, #B6157)) were added. Finally, cells were lysed with 0.2% saponin and the lysate was plated on agar plates for counting.

### Indirect immunofluorescence

The macrophages (5 × 10^4^ cells/well) were cultured in 24-well plates for 24 h before being treated according to gentamicin protection assay. Cells were fixed with 4% paraformaldehyde at room temperature (RT) for 30 minutes, followed by permeabilization with 0.02% Triton X-100 for 10 minutes. To block nonspecific binding, cells were incubated with 4% goat serum in PBS for 30 minutes. The cells were incubated with anti-LC3 (Sigma-Aldrich, #SAB5701328) at RT for 1 h and washed three times with PBS, followed by incubation with Texas Red fluorescent secondary antibody for 1 h. Subsequently, cells were incubated with anti-*S.* Typhimurium (Abcam, #Ab35156) for 1 h. After three washes with PBS, cells were then incubated with an Alexa Fluor^®^ 488-conjugated secondary antibody for 1 h. Nuclei were stained with Hoechst dye. Fluorescence signals were observed under a fluorescence microscope (Olympus IX-83). The analysis and quantification of LC3B puncta were performed using confocal microscopy.

### Determination of minimum inhibitory concentration

Determination of the MIC of PHZ on *S.* Typhimurium used the broth microdilution according to the standard procedure in microbiology and pharmacology research. PHZ was two-fold serially diluted from 64 to 0.0625 μg/mL in a 96-well plate. Subsequently, each well was supplemented with LB medium containing *S.* Typhimurium (5 × 10^6^ CFUs/mL) and 0.02% (w/v) resazurin, establishing a standardized microdilution system for minimum inhibitory concentration (MIC) determination. The plate was incubated at 37°C for 18 h. The MIC was defined as the lowest concentration of PHZ at which the resazurin reagent remained blue (indicating no bacterial growth), in contrast to the pink color change that occurs in wells with bacterial growth.

### Cell viability assay

Macrophages (10^5^ cells/well) were grown in 24-well plates for 24 h, followed by PHZ treatment for 24 h. Cell viability was determined using a Cell Counting Kit-8 (CCK-8) (Beyotime, #C0039), according to manufacturer’s instructions.

### Secretion assay of *S.* Typhimurium SPI-1 T3SS effectors

High concentration of salt induced the secretion of *S.* Typhimurium T3SS effectors *in vitro*. The overnight culture of *S.* Typhimurium was inoculated into LB broth (0.5M NaCl) containing the corresponding concentration of PHZ at a 1:50 ratio. After 4 h of culture, culture supernatants were collected and incubated with trichloroacetic acid (TCA) overnight at 4°C as described previously (26). The precipitates were resuspended in 1×SDS loading buffer after centrifugation at 12,000 × g for 20 min. The effectors were determined by Coomassie brilliant blue (CBB) staining and western blotting.

### RNA isolation, library preparation and sequencing

Raw 264.7 cells were treated as described in the preceding section for infection and drug treatment. Total RNA from the cells was isolated using TRIzol™ reagent (Thermo Fisher, #15596018) according to the manufacturer’s instructions. Then, the purity and integrity of the extracted RNA were assessed using NanoDrop ND-1000 (NanoDrop, Wilmington, DE, USA) and its integrity was checked by Bioanalyzer 2100 (Agilent, CA, USA). Samples that met the quality inspection (concentration >50 ng/μL, RNA integrity number (RIN) value >7.0, and total RNA >1μg) were used for preparing cDNA libraries. Finally, paired-end sequencing (PE150) was performed on the Illumina NovaSeq 6000 platform (LC-Bio Technology Co., Ltd., Hangzhou, China) following the manufacturer’s standard protocols. The threshold for selecting DEGs were set at a fold change (FC) of ≥2 or <0.5 (absolute value of log2FC ≥ 1), and the false discovery rate (FDR) is controlled to be less than 0.05 to screen DEGs. Bioinformatics analysis is performed using the OmicStudio tool.

### Quantitative reverse transcription PCR

Reverse transcription of mRNA to cDNA was carried out using RevertAid RT reverse transcription kit (Thermo Fisher, #K1691). RT-PCR was performed using SYBR Green master mix (AlpaLife, #KTSM1401) on an Applied Biosystems Real-Time PCR System (Quantstudio 1; Thermo Fisher). All primers used in this study were listed in **Table 1**. The relative gene expression levels were assessed using the 2^-ΔΔCt^ method.

**Table 1:** Primes used for RT-PCR.

Primer name	Sequence (5′-3′)
*gpnmb-*F	CCCTCTGAGAGCAGTGAATGG
*gpnmb-R*	GTTCCTGGGGCAGTTTCCTAT
*sqstm1-*F	GATAGCCTTGGAGTCGGTGG
*sqstm1-R*	CCGGGGATCAGCCTCTGTAG
*mpeg1-*F	CAACTCCAGGGTGCAGAGTT
*mpeg1-R*	GCCAGGTAGCTTGTCAGGTT
*creg1-*F	ATCCAGAGGCTACGCTGACT
*creg1-R*	CTTGGTCACAGTTCCCGACA
*tcirg1-*F	TCTCCAACACAGCCTCCTACT
*tcirg1-R*	CAGTCAACACAGCAAAGGCA
*gpr137b-*F	CCAGCCATGGATTCAGTCCC
*gpr137b -R*	GCAAAGTTCCTGCTTGTGCC
*slc25a37-*F	GCTAATGGGGTAGCTGGGAG
*slc25a37-R*	TGTCCGGATACAACTGAAGGC
*slc31a2-*F	GGCAAAGCCAAATTGCTCCA
*slc31a2-R*	GAGGCGGGTCCTATTGTCTG
*cyb5r1-*F	GGGATCCAGCCGGTTACTCT
*cyb5r1-R*	AGACTGCCATCAATTCGGGC
*glipr1-*F	GATAGTCTGGATGGCTTCGT
*glipr1-R*	CTCACTTTTGACCGAAGCTG
*odc1-*F	TGTGGGTGATTGGATGCTGTT
*odc1-R*	GGATCTGCTTCATGAGTTGCCA
*ccl2-*F	GACCCCAAGAAGGAATGGGT
*ccl2-R*	ACCTTAGGGCAGATGCAGTT
*dad2-*F	AGGCCCTAATGACCCTTGATG
*dad2-R*	GTCGTTTGCTGAAGATGTTGGA
*ptgs2-*F	CATCCCCTTCCTGCGAAGTT
*ptgs2-R*	GGCCCTGGTGTAGTAGGAGA
*socs3-*F	GTTGAGCGTCAAGACCCAGT
*socs3-R*	GAGTACACAGTCGAAGCGGG
*sfta-*F	AAGTCTTCAGAGGCCTTTCTGG
*sfta-R*	ATGGAGGTCAGCTCATCAGT
*nfκb-*F	CGTTCCTGCACTTGGCAATC
*nfκb-R*	TCAGCAATTCCTGGCTGGTT
*glpx-*F	GTGGTCGTGTTCATCAAGCC
*glpx-R*	CGCACTGGTGTTGTTAGTGG
*cstdc4-*F	CCAGGAGATTGCTGATAAGGTTAAG
*cstdc4-R*	TCCAGCGACGACTTGAGATT
*csta3-*F	GGAGATTGCTAACAAGGTCAGA
*csta3-R*	CCAGAATGTGCACGGTAGACT

### Measurement of ROS

Intracellular ROS generation in macrophages was evaluated according to the fluorescence intensity of dihydroethidium (DHE) probe. Macrophages (10^4^ cells/well) were grown in 96-well plates for 24 h, then were treated according to the gentamicin protection assay. ROS in macrophages were detected under a fluorescence microscope (Olympus IX-83) after co-incubation with 5 μM DHE (Beyotime, #S0063) for 30 min and quantified by Image J software.

### Examination of lysosomal acidification

Macrophages (10^4^ cells/well) were grown in 96-well plates for 24 h, then were treated according to the gentamicin protection assay. The lysosomal acidification was assessed using LysoTracker and Lysosensor according to the manufacturer’s instructions. Fluorescence was visualized under a fluorescence microscope (Olympus IX83) and quantified using ImageJ software.

### ATP detection

Macrophages (10^6^ cells/well) were grown in 6-well plates for 24 h, then were treated according to the gentamicin protection assay. The ATP level was measured using Enhanced ATP Assay Kit (Beyotime, #S0027) under a full-wavelength microplate reader (Agilent BioTek Synergy H1), according to manufacturer’s instructions.

### Western blot

Cells were lysed by NP-40 lysis buffer containing protease inhibitor and protein concentration was quantified by BCA protein assay kit according to the manufacturer. Equal amounts of protein were separated by SDS-PAGE and transferred onto PVDF membranes. Proteins were recognized by primary antibodies against LC3 (Sigma, #SAB5701328), p62 (Abcam, #ab109012), GAPDH (ABclonal, #A19056), Tubulin (Abcam, #ab210797), SipA, and ICDH (Sigma, #abs2090) and detected using fluorescence dye-conjugated secondary antibodies under the Odyssey CLX Imaging System (Li-Cor).

### Animal experiments

Female BALB/c mice (6–8 weeks old, approximately 20 g) were purchased from Liaoning Changsheng Biotechnology Co., Ltd. (Benxi, China). Mice were bred in ventilated cage with a stable temperature of 23 ± 2°C and a humidity of 55% under a 12-h light/dark cycle. After an acclimatization period at least one week, mice received streptomycin (5 g/L) in their drinking water for 3 days before *S.* Typhimurium infection. Mice were randomly divided into three groups (n=6): DMSO (Blank control), *S.* Typhimurium (the infection control), and *S.* Typhimurium + PHZ (PHZ treatment). Mice were infected with 5 × 10^6^ CFUs intragastrically. Mice in the PHZ treatment group were administered 1mg/kg PHZ subcutaneously once every day. All infected mice were euthanized 4 days post-infection. The liver, spleen, and colon (with cecum) were aseptically collected. One part of these tissues was homogenized in PBS and plated on LB agar plates containing 50 μg/mL streptomycin for CFU counting. Cytokine levels in homogenate of tissues was measured using specific ELISA accordingly to the manufacturer’s instruction. Another part of these tissues was fixed in 4% paraformaldehyde. Hematoxylin and eosin (H&E) staining was performed on 4-5 μm paraffin-embedded sections. All mouse experiments were approved by Jilin University Institutional Animal Care Committee and strictly conducted according to the guidelines of this committee (SY202412052).

### Statistical analysis

The intensity of western blot bands and the fluorescence intensity of LysoTracker, LysoSensor and DHE were quantified using ImageJ (NIH, Bethesda, MD, USA). All statistical analyses were performed using GraphPad Prism 9.5 (GraphPad Software, USA). Two independent groups were compared using an unpaired two-tailed Student’s *t*-tests; three or more groups were compared using Tukey’s honestly significant difference (HSD) test following one-way ANOVA implementation. *In vivo* survival rates were assessed with the log-rank (Mantel–Cox) test. Data are shown as mean ± SD. P-values in the figures are labeled as follows: ns, not significant; ***P < 0.001; **P < 0.01; and *P < 0.05.

## Results

### PHZ enhances antibacterial activity of macrophages

**Figure 1:**
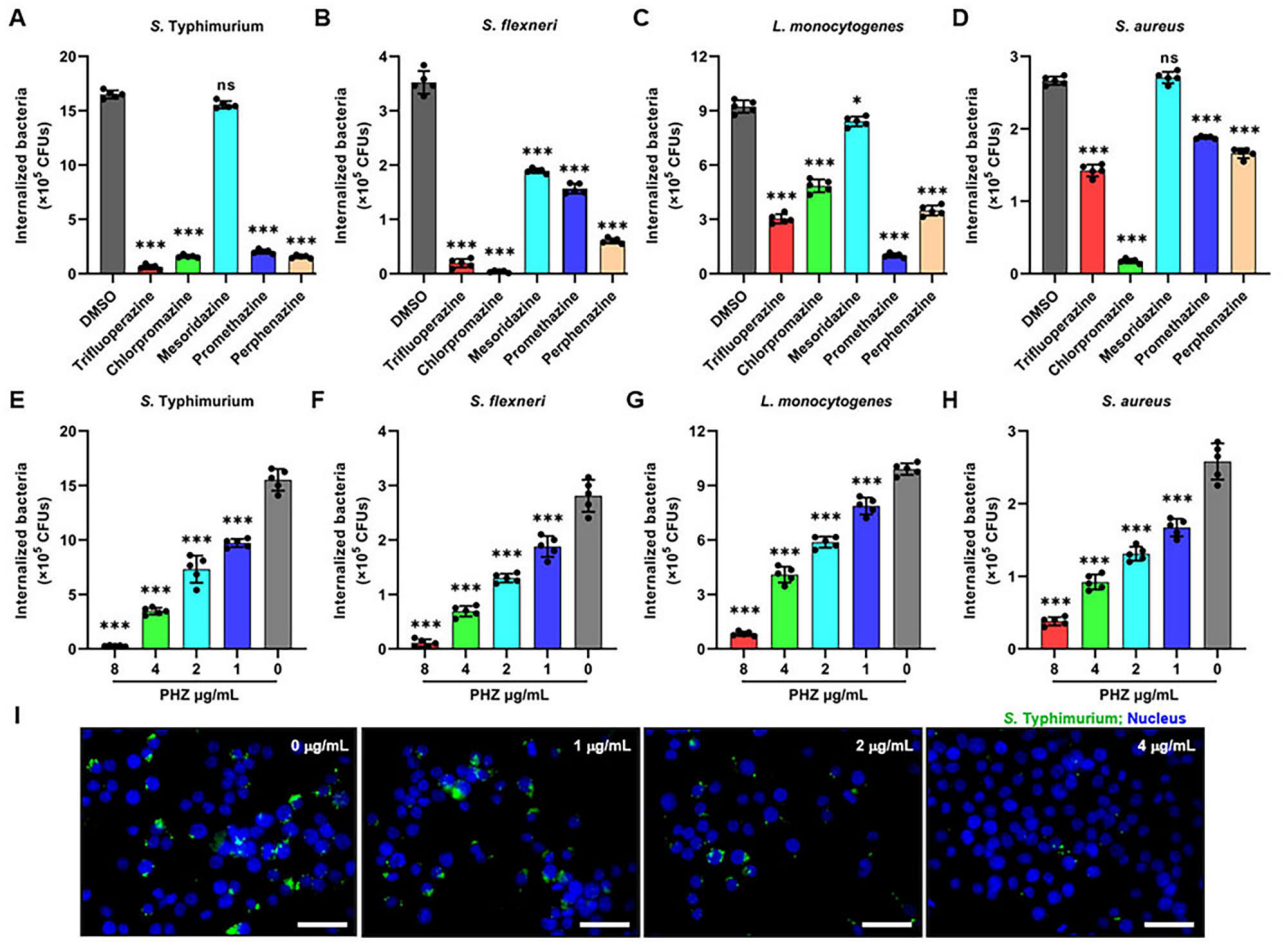
Phenothiazines enhances macrophage antimicrobial activity. **(A-D)** Raw 264.7 cells infected with *Salmonella enterica* serovar Typhimurium (*S.* Typhimurium) **(A)**, *Shigella flexneri* (*S. flexneri*) **(B)**, *Listeria monocytogenes (L. monocytogenes*) **(C)** or *Staphylococcus aureus* (*S. aureus*) **(D)** were incubated with indicated phenothiazine (4 μg/mL) and gentamicin for 8 hours and CFUs were quantified **(E-H)** Gentamicin protection assay on Raw 264.7 cells with different bacteria in the presence of perphenazine (PHZ). Macrophages were infected for 30 mins with *Salmonella enterica* serovar Typhimurium (*S.* Typhimurium) **(E)**, *Shigella flexneri* (*S. flexneri*) **(F)**, *Listeria monocytogenes* (*L. monocytogenes*) **(G)** or *Staphylococcus aureus* (*S. aureus*) **(H)** followed by gentamicin treatment with increasing concentrations of PHZ (0-8 μg/mL) for 8 h before cell lysis. Values represent absolute CFU counts. Every data point represents an independent experimental replicate. **(I)** Immunofluorescence of intracellular *S.* Typhimurium (Green) on Raw 264.7 cells with increasing concentrations of PHZ (0-4μg/mL), Hoechst (blue) was used to visualize nuclei (scale bar: 20 μm). Data presented in panels A-H are represented as the mean ± SD, n = 5. ns indicates P > 0.05, * indicates P < 0.05 and *** indicates P < 0.001 by one-way ANOVA. Panel **(I)** is representative of five independent experiments.

To assess the impact of phenothiazines on the antibacterial activity of macrophages, we measured the intracellular replication of a range of bacteria including *S.* Typhimurium, *S. flexneri*, *S. aureus* and *L. monocytogenes*, using a gentamicin protection assay in the presence or absence of phenothiazines in Raw 264.7 cells. 4 μg/mL of phenothiazines (promethazine, trifluoperazine, chlorpromazine and perphenazine), other than mesoridazine, significantly inhibited the replication of all the above-mentioned bacteria in macrophages (**Figures 1A–D**). Based on the shared chemical structure of phenothiazines and the above experimental results, we speculated that phenothiazines may inhibit the replication of bacteria in macrophages through a common mechanism. Therefore, we selected perphenazine (PHZ) as a representative phenothiazine for further investigation. PHZ markedly inhibited distinct intracellular bacterial replication in a dose-dependent manner in macrophages, even at a concentration as low as 1 μg/mL (**Figures 1E–H**). Consistently, immunofluorescent staining confirmed that PHZ eliminated the intracellular *S.* Typhimurium in macrophages (**Figure 1I**). To ascertain whether the inhibition of intracellular *S.* Typhimurium replication in macrophages by PHZ is mediated through suppression of the host cell proliferation or direct antibacterial activity, we evaluated the effects of PHZ on macrophage viability and bacterial virulence. PHZ did not cause macrophages death at 8 μg/mL over an incubation period of 24 h (**Supplementary Figure S1A**). Additionally, since the replication of *S.* Typhimurium in macrophage is intricately associated with both the vitality of bacteria and the secretion ability of T3SS, we investigated whether PHZ has an impact on the replication and the secretion of SPI-1 T3SS effectors of *S.* Typhimurium *in vitro*. The MIC of PHZ against *S.* Typhimurium exceeded 64 μg/mL (**Supplementary Figure S1B**). PHZ had no effect on the expression and secretion of SPI-1 T3SS effectors in *S.* Typhimurium culture supernatant (**Supplementary Figure S1C-E**). Taken together, these data indicated that PHZ enhances the antibacterial ability of macrophages without compromising cell viability or bacterial pathogenicity.

### PHZ modulates the transcriptome of naive and *S.* Typhimurium-infected macrophages

**Figure 2:**
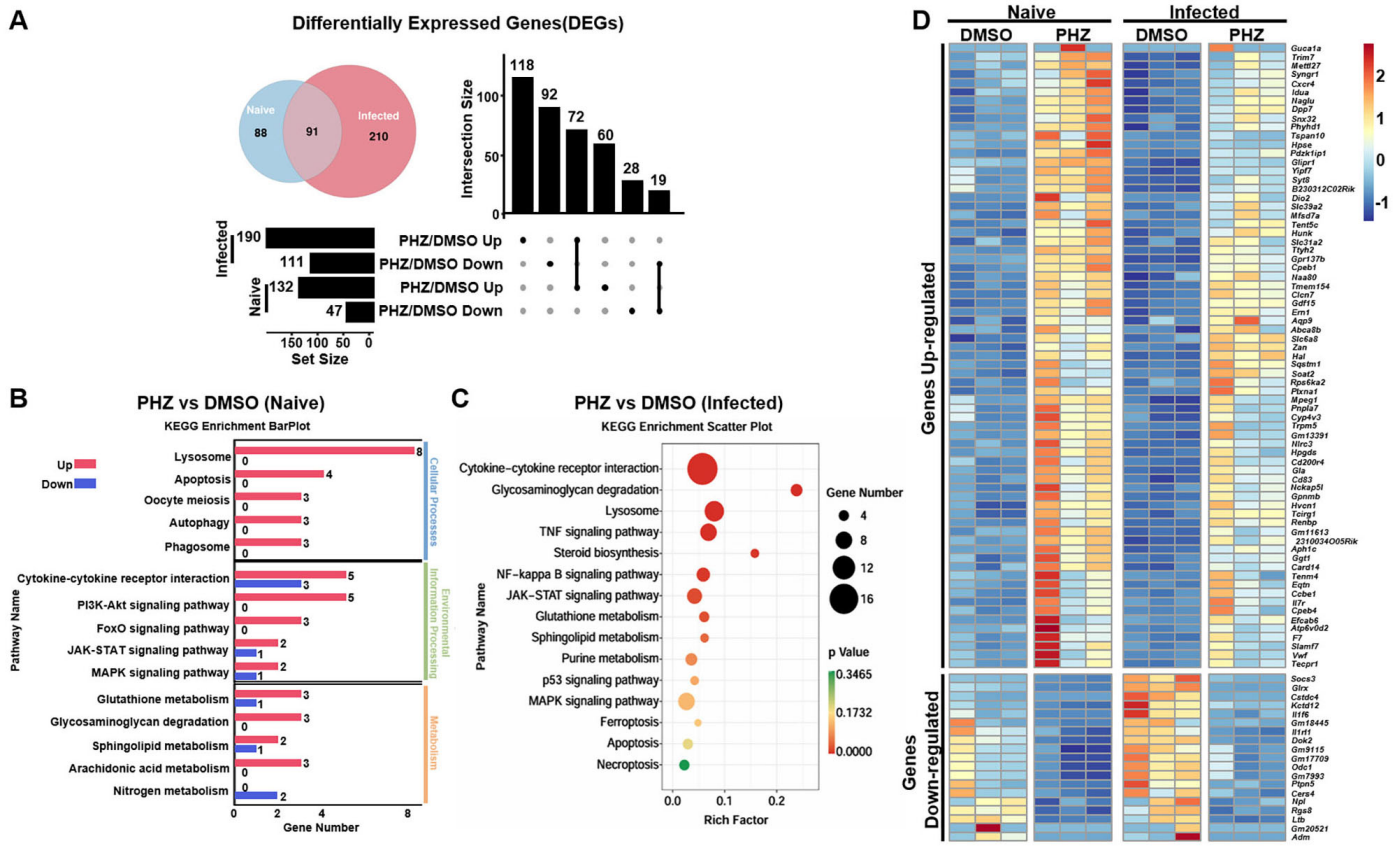
PHZ modulates the transcript of Raw 264.7 cells. **(A)** Venn diagram of the number of differentially expressed genes induced by PHZ in naive (blue) and *S.* Typhimurium-infected (red) macrophages. The bar graph displays the number of differentially expressed genes upregulated or downregulated following PHZ treatment in either untreated or *S.* Typhimurium -infected macrophages. **(B)** Raw 264.7 cells were treated with PHZ (4 μg/mL) for 18 h, KEGG pathway enrichment of differentially expressed genes affected by PHZ in naive cells. **(C)** Raw 264.7 cells were infected with *S.* Typhimurium at MOI = 10 for 30 mins and then treated with PHZ (4 μg/mL) and gentamicin for an additional 18 h, KEGG pathway enrichment of differentially expressed genes by PHZ in KEGG pathway enrichment of differentially expressed genes affected by PHZ in *S.* Typhimurium-infected cells. **(D)** Heatmap of differential expression of genes induced by PHZ in naive and *S.* Typhimurium-infected cells. Each column represents an independent experimental replicate. Data were normalized to determine the log ratio with respect to the median expression of each gene.

Based on the above results, we further hypothesized that PHZ may inhibit bacterial intracellular replication by modulating the cellular processes of macrophages. RNA sequencing (RNA-seq) was used to analyze the effect of PHZ on the genome-wide transcription levels of macrophages. In naive cells, compared with the DMSO-treated group (Blank Control), the transcriptional levels of 179 genes were significantly altered following PHZ treatment, with 132 genes upregulated and 47 genes downregulated (**Figure 2A**). *S.* Typhimurium infection significantly altered the transcription of 301 genes, comprising 190 upregulated and 111 downregulated genes (**Figure 2A**). Compared with the number of differentially expressed genes (DEGs) induced by PHZ in naive macrophages, a greater number of DEGs were observed in *S.* Typhimurium-infected macrophages following PHZ treatment, which may be attributed to the transcriptional reprogramming of macrophages induced by *S.* Typhimurium infection. Furthermore, 91 genes displayed significant transcriptional changes in both naive and *S.* Typhimurium-infected macrophages induced by PHZ (**Figures 2A, D**), suggesting a common regulatory response to PHZ treatment across *S.* Typhimurium infection. To validate the accuracy of the RNA-seq data, we conducted RT-PCR analysis on a subset of 20 DEGs. The results revealed that the transcription levels of 10 genes were upregulated following infection but downregulated after PHZ treatment, whereas the remaining 10 genes exhibited downregulation after infection and upregulation upon PHZ treatment (**Supplementary Figure S2**). These were consistent with those obtained from the transcriptome profiling, thereby substantiating the reliability and accuracy of the RNA-seq dataset. Kyoto Encyclopedia of Genes and Genomes (KEGG) enrichment analysis of the DGEs induced by PHZ revealed that a significant number of DEGs were associated with lysosomal and glycosaminoglycan degradation in naive or *S.* Typhimurium-infected macrophages (**Figures 2B,C**). Moreover, compared to naive cells, DEGs induced by PHZ in *S.* Typhimurium-infected cells were predominantly enriched in the cytokine–cytokine receptor interaction, with the majority of these genes exhibiting significantly downregulated transcription levels (**Figure 2C**). The transcriptional alterations in macrophages induced by PHZ may suppress the intracellular replication of *S.* Typhimurium.

### PHZ enhances macrophage lysosomal activity

**Figure 3:**
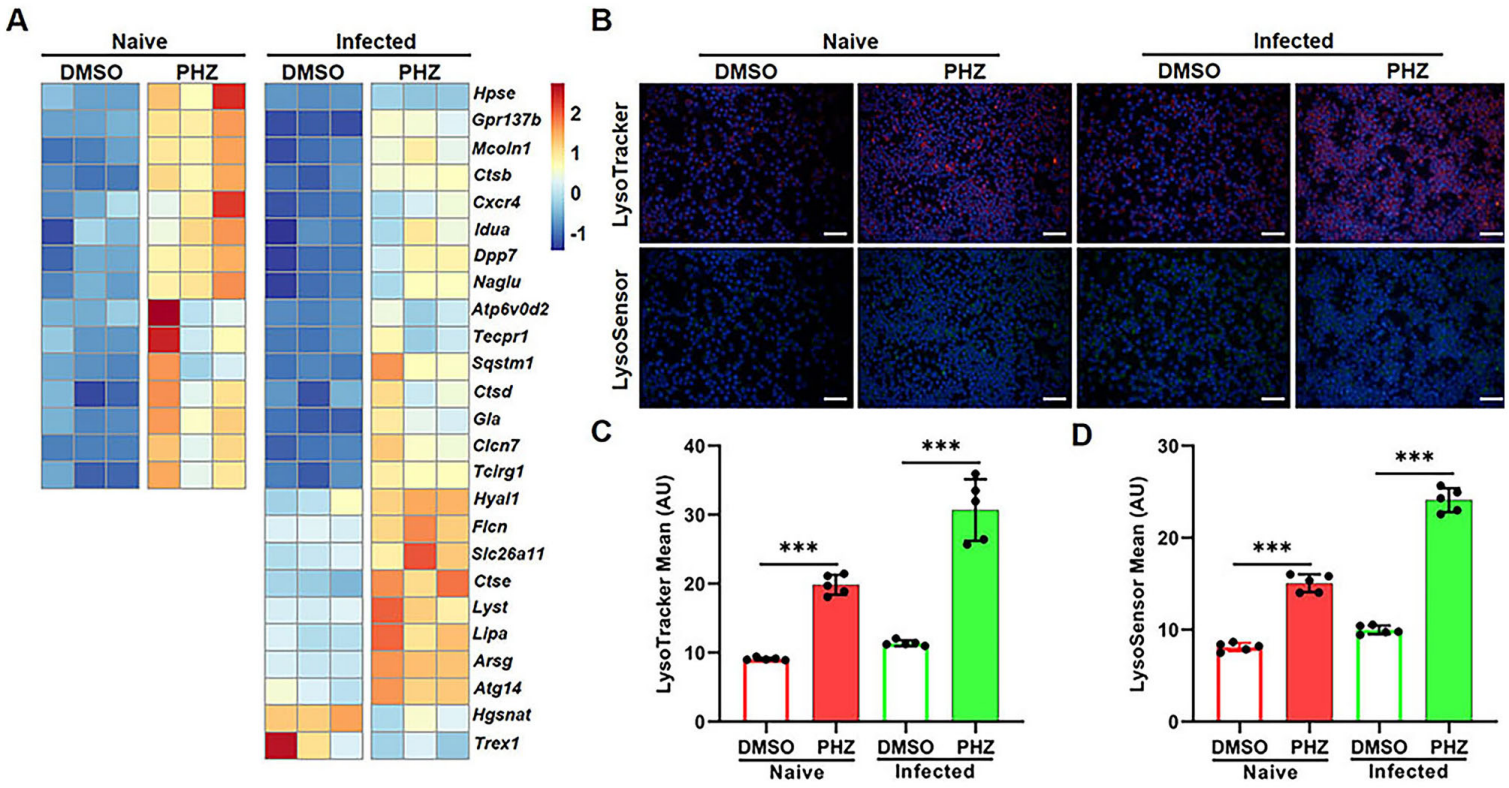
PHZ activates the lysosomal pathway in *S.* Typhimurium-infected macrophages. **(A)** Heatmap of differential expression of genes included in the Lysosome KEGG category. Each column represents an independent experimental replicate. Data were normalized to determine the log ratio with respect to the median expression of each gene. **(B-D)** Raw 264.7 cells were infected with *S.* Typhimurium at MOI = 10 for 30 mins and then treated with PHZ (4 μg/mL) and gentamicin for an additional 8 h After 8 h of treatment, cells were labelled with LysoTracker (red) and LysoSensor (green), then fluorescence was analyzed by fluorescence microscopy **(B)**. Hoechst (blue) was used to visualize nuclei (scale bar: 50 μm). The quantification of LysoTracker staining **(C)** and LysoSensor staining **(D)** were performed using Image J software. Data presented in panels **(C, D)** are represented as the mean ± SD, n = 5. *** indicates *P* < 0.001 by one-way ANOVA. Panel **(B)** is representative of five independent experiments.

Lysosomes are acidic organelles containing various hydrolases, and are involved in innate immune responses and cellular homeostasis by detecting and eliminating microbes and debris in macrophages (27, 28). Additionally, lysosomes function as essential signal transduction platforms, capable of sensing and responding to alterations in cellular stress and nutritional status (29, 30). The preservation of normal lysosomal function critically relies on three distinct classes of proteins: lumenal proteins, integral membrane proteins, and membrane-associated proteins (31, 32). Based on the KEGG database, functional analysis of DEGs demonstrated that PHZ affects lysosomal function (**Figures 2B,C**). In both naive and *S.* Typhimurium-infected macrophages, we identified 15 and 25 lysosomal-related DEGs, respectively (**Figure 3A**). With the exception of a small subset of integral membrane proteins and membrane-associated proteins, these genes predominantly encode hydrolases and V-type ATPase subunits, and their transcriptional levels were significantly upregulated. Transcriptomic analysis indicated that PHZ may enhance lysosomal activity. To validate this hypothesis, we employed LysoTracker and LysoSensor to label and assess lysosomes in macrophages. Compared with naive cells, fluorescence intensity did not change significantly in *S.* Typhimurium-infected cells. However, following PHZ treatment, the fluorescence intensities of lysosomes labeled with LysoTracker and LysoSensor were both markedly increased (**Figures 3B–D**). These findings indicated that PHZ treatment significantly increased lysosomal acidity in macrophages. Existing studies have demonstrated that the acidic environment within lysosomes enhances the activity of hydrolases (30). Consequently, these results further support the notion that PHZ treatment enhances the lysosomal activity in macrophages.

### PHZ induces autophagy in macrophages

**Figure 4:**
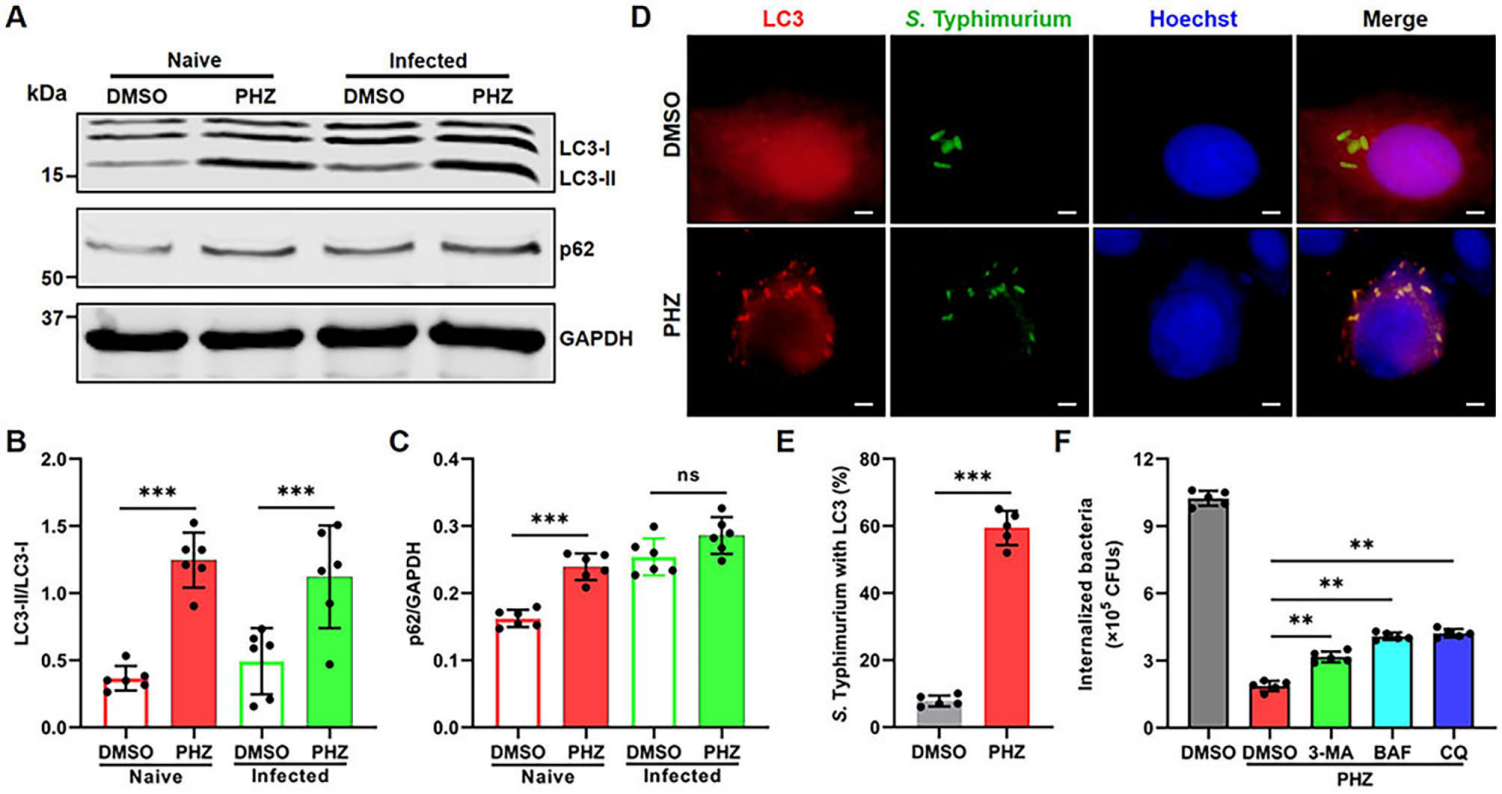
PHZ induces autophagy in macrophages. **(A-C)** Representative immunoblot of the expression of LC3, p62, and GAPDH in naive and *S.* Typhimurium-infected cells treated with PHZ (4 μg/mL) **(A)**. Protein quantification performed by ImageJ of LC3-II compared to LC3-I **(B)** and p62 compared to GAPDH **(C)**. **(D)** Detection by indirect immunofluorescence of LC3 (red) in *S.* Typhimurium (green) infected macrophages, treated with PHZ (4 μg/mL) for 8 h (scale bar: 5 μm). Hoechst (blue) was used to visualize nucleus. **(E)** Quantitation of LC3 enriched with *S.* Typhimurium. At least 100 bacteria were counted for each infection, and the data shown are the mean ± SD of five independent assays. **(F)** Gentamicin protection assay performed on control macrophages, PHZ macrophages, and PHZ macrophages treated with the 3-MA (20 mM), BAF (bafilomycin A1) (20 nM) or CQ (chloroquine) (20 μM) for 8h. Data presented in panels **(B, C, E, F)** are represented as the mean ± SD, n = 5. ns indicates *P* > 0.05, ** indicates *P* < 0.01 and *** indicates *P* < 0.001 by one-way ANOVA **(B, C, F)** or Student’s *t*-test **(E)**. Panel **(A)** and panel **(D)** are representative of five independent experiments.

Autophagy is a highly conserved catabolic process that mediates the degradation of unnecessary or dysfunctional cellular components via lysosomes, including long-lived proteins, misfolded proteins, damaged organelles, and invading pathogenic microbes, thereby playing a pivotal role in maintaining cellular homeostasis (14, 33). Accumulating evidence has demonstrated a strong correlation between lysosomal activity and the progression of autophagy (34, 35). Given the established role of PHZ in promoting lysosomal acidification, we hypothesized that PHZ may induce autophagy in macrophages. To test this hypothesis, we used immunoblot and immunofluorescence assays to detect lipidated microtubule-associated protein light-chain 3 (LC3-II), which is specifically recruited to the autophagosomal membranes and serves as a critical marker of autophagy (36). Immunoblot analysis revealed that PHZ treatment significantly increased the levels of LC3-II in naive and *S.* Typhimurium-infected macrophages (**Figures 4A,B**). Consistently, we observed an increase in LC3 puncta per cell after PHZ treatment by fluorescence microscopy (**Supplementary Figure S3**). In addition, we found that phenothiazines (promethazine, trifluoperazine, chlorpromazine and perphenazine), other than mesoridazine, markedly enhanced the accumulation of LC3-II within macrophages (**Supplementary Figure S4**). p62 (SQSTM1) is a ubiquitin-binding scaffold protein that is selectively degraded by autophagy, making it a widely utilized marker for assessing autophagic flux (27, 37). Notably, PHZ treatment resulted in increased p62 protein levels in naive macrophages (**Figures 4A, C**), which may be due to the increased transcription of p62 induced by PHZ (**Supplementary Figure S2**). In *S.* Typhimurium-infected macrophages, immunofluorescence analysis indicated that the proportion of intracellular *S.* Typhimurium colocalized with LC3 puncta increased significantly following PHZ treatment (**Figures 4D,E**). This finding suggested that PHZ may enhance the clearance of intracellular *S.* Typhimurium by promoting autophagy. Thus, we evaluated the effect of three autophagy inhibitors on PHZ-mediated suppression of intracellular bacterial replication: bafilomycin A1 (BAF, a lysosomal v-ATPase inhibitor), chloroquine (CQ, which blocks endosomal/lysosomal acidification and autophagosome-lysosome fusion), and 3-methyladenine (3-MA, a type III PI3K inhibitor that prevents autophagosome formation) (38). All three inhibitors partially attenuated the antibacterial effect of PHZ, though the extent of reversal was relatively modest (**Figure 4F**). Collectively, these results indicate that PHZ partially inhibits the intracellular replication of S. Typhimurium by inducing autophagy.

### PHZ modulates glucose metabolism and induces the accumulation of ROS in macrophages

**Figure 5:**
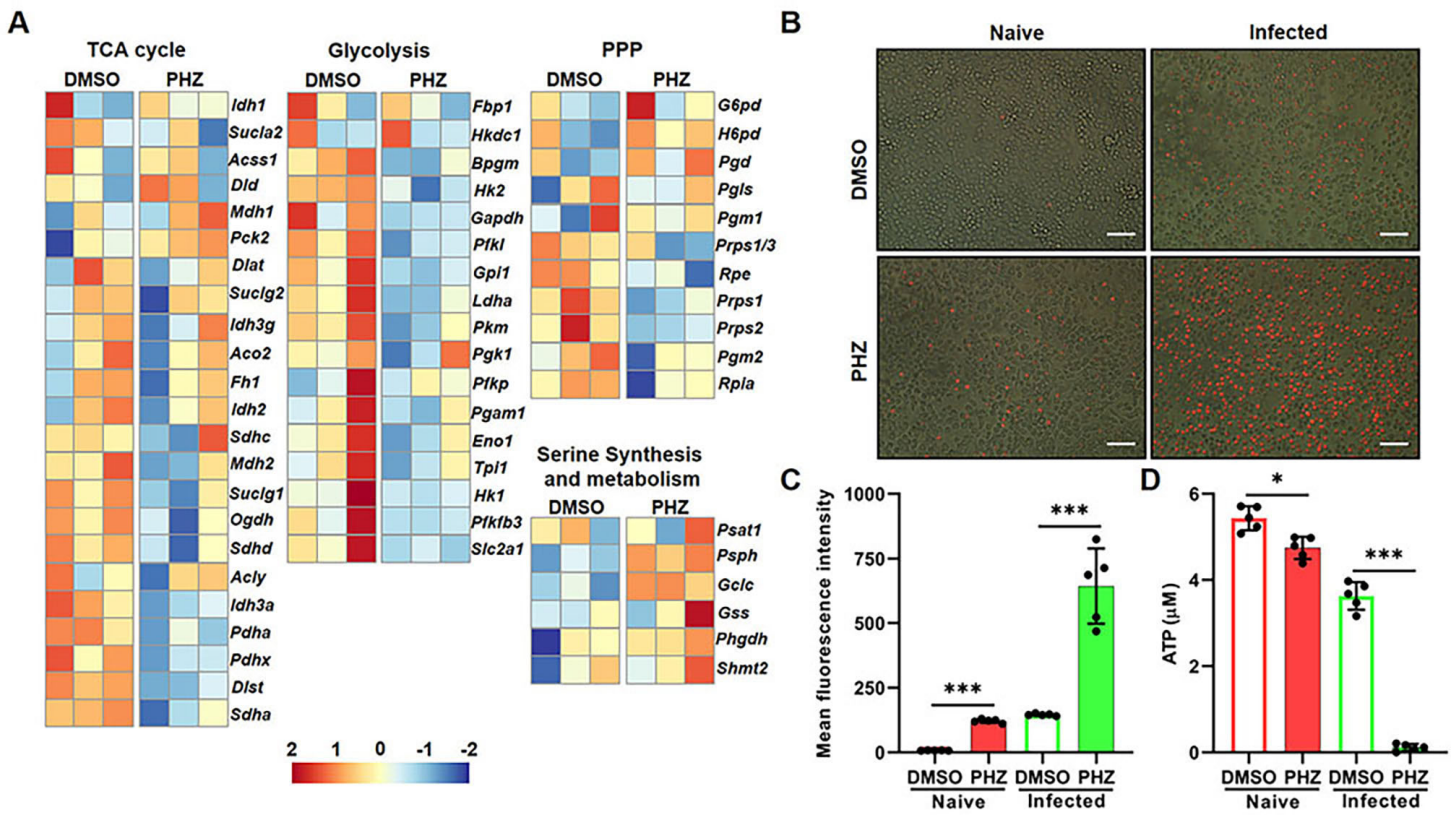
PHZ modulates glucose metabolism and induces the accumulation of ROS in *S.* Typhimurium-infected macrophages. **(A)** Heatmap of the expression profiles of glucose metabolism genes in *S.* Typhimurium-infected cells treated with PHZ (4 μg/mL). Each column represents an independent experimental replicate. Data were normalized to determine the log ratio with respect to the median expression of each gene. **(B, C)** Raw 264.7 cells were infected with *S.* Typhimurium at MOI = 10 for 30 mins and then treated with PHZ (4 μg/mL) and gentamicin for an additional 8 h After 8 h of treatment, cells were labelled with DHE (red), then fluorescence was analyzed by confocal microscopy **(B)** (scale bar: 50 μm). The quantification of DHE staining **(C)** were performed using Image J software. **(D)** The ATP level in naive and *S.* Typhimurium-infected cells following PHZ treatment. Data presented in panels **(C, D)** are represented as the mean ± SD, n = 5. * indicates *P* < 0.05 and *** indicates *P* < 0.001 by one-way ANOVA. Panel **(B)** is representative of five independent experiments.

Based on the aforementioned findings, we proposed that PHZ might elicit cellular responses other than autophagy in macrophages to limit the intracellular replication of *S.* Typhimurium. KEGG enrichment analysis revealed that DEGs induced by PHZ were enriched not only in the lysosomal pathway but also in several key metabolic pathways, including glutathione metabolism, nitrogen metabolism, lipid metabolism, and arachidonic acid metabolism (**Figure 2B**). These findings suggested that PHZ modulates macrophage metabolism. Previous studies have demonstrated that glucose starvation can trigger autophagy (39). Analysis of glucose metabolism-related genes in the transcriptome showed that the transcriptional levels of genes associated with the tricarboxylic acid (TCA) cycle and glycolysis were significantly reduced following PHZ treatment in naive and *S.* Typhimurium-infected macrophages (**Figure 5A**; **Supplementary Figure S5**). Likewise, the ATP level in macrophages treated with PHZ was significantly reduced, particularly under *S.* Typhimurium infection (**Figure 5D**). A reduction in ATP levels is commonly associated with the accumulation of intracellular ROS. Compared to the DMSO-treated group (Blank Control), intracellular ROS levels as stained by dihydroethidium (DHE) were significantly elevated in macrophages following infection with *S.* Typhimurium, an effect mediated by bacterial lipopolysaccharide (LPS) (**Figures 5B,C**). In naive cells, intracellular ROS levels showed only a modest increase following PHZ treatment. In contrast, in *S.* Typhimurium-infected cells, PHZ treatment led to a marked elevation in intracellular ROS levels (**Figures 5B,C**). These findings indicated that PHZ promotes ROS accumulation in macrophages, with a significantly enhanced effect observed in the context of *S.* Typhimurium infection. In addition, phenothiazines (promethazine, trifluoperazine, chlorpromazine and perphenazine), other than mesoridazine, promoted the accumulation of ROS in *S.* Typhimurium*-*infected macrophages (**Supplementary Figure S6**). Taken together, PHZ effectively modulates glucose metabolism and promotes the accumulation of ROS in macrophages.

### ROS induced by PHZ eliminates intracellular bacteria and promotes autophagy in macrophages

**Figure 6:**
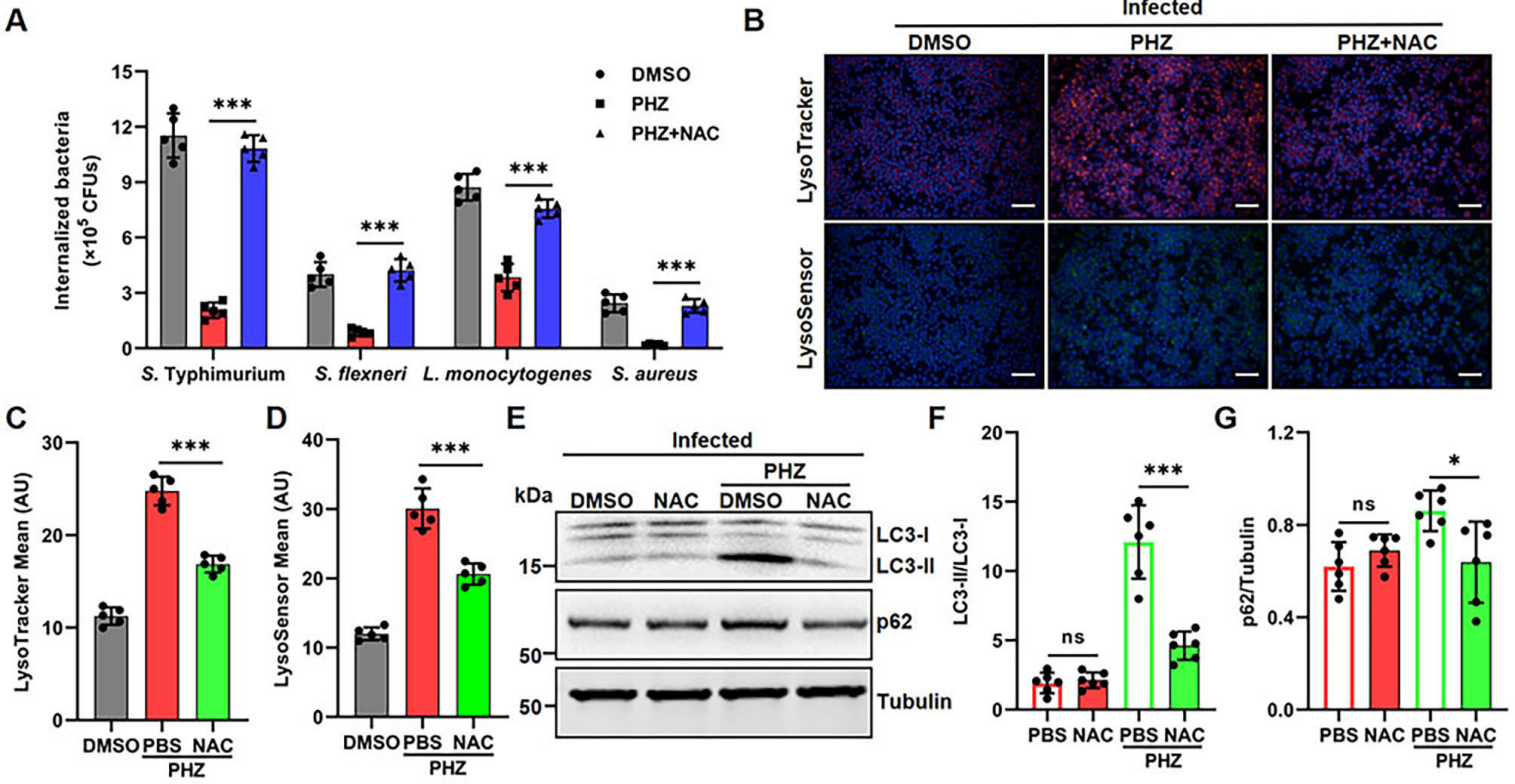
ROS contribute to PHZ-induced autophagy in macrophages. **(A)** Gentamicin protection assay performed on control macrophages, PHZ macrophages, and PHZ macrophages treated with NAC (5mM) for 8h. **(B-D)** Raw 264.7 cells were infected with *S.* Typhimurium at MOI = 10 for 30 mins and then treated with PHZ (4 μg/mL) and gentamicin for an additional 8 **(h)** After 8 h of treatment, cells were labelled with LysoTracker (red) and LysoSensor (green), then fluorescence was analyzed by fluorescence microscopy **(B)**. Hoechst (blue) was used to visualize nuclei (scale bar: 50 μm). The quantification of LysoTracker staining **(C)** and LysoSensor staining **(D)** were performed using Image J software. **(E-G)** Representative immunoblot of the expression of LC3, p62, and tubulin in *S.* Typhimurium-infected cells treated with or without PHZ (4 μg/mL) and NAC (5mM) **(E)**. Protein quantification performed by ImageJ of LC3-II compared to LC3-I **(F)** and P62 **(G)** compared to tubulin. Data presented in panels **(C-D, F-G)** are represented as the mean ± SD, n = 5. * indicates *P* < 0.05 and *** indicates *P* < 0.001 by one-way ANOVA. Panel **(B)** is representative of five independent experiments.

Accumulating evidence demonstrates that ROS produced by macrophages can effectively eliminate intracellular bacteria through the oxidative damage of bacterial DNA, lipids, and proteins (40–42). To further explore the mechanism through which PHZ facilitates the elimination of intracellular bacteria, we proposed that ROS induced by PHZ plays an essential role in this process. Our results indicated that the supplementation of NAC, an exogenous ROS scavenger, markedly suppressed the clearance of intracellular bacteria when PHZ was present (**Figure 6A**). Moreover, it has been shown that ROS produced by NADPH oxidase (NOX) are capable of inducing cellular autophagy activation, which contributes to their antibacterial effects (41, 43). We next assessed whether ROS were involved in PHZ-induced autophagy. In *S.* Typhimurium-infected macrophages, the addition of NAC significantly attenuated the lysosomal acidification and the accumulation of LC3-II induced by PHZ treatment (**Figures 6B–G**). These findings indicate that the elevation of ROS following PHZ treatment contributes to the clearance of intracellular bacteria and the induction of autophagy.

### PHZ protects mice from *S.* Typhimurium infection

**Figure 7:**
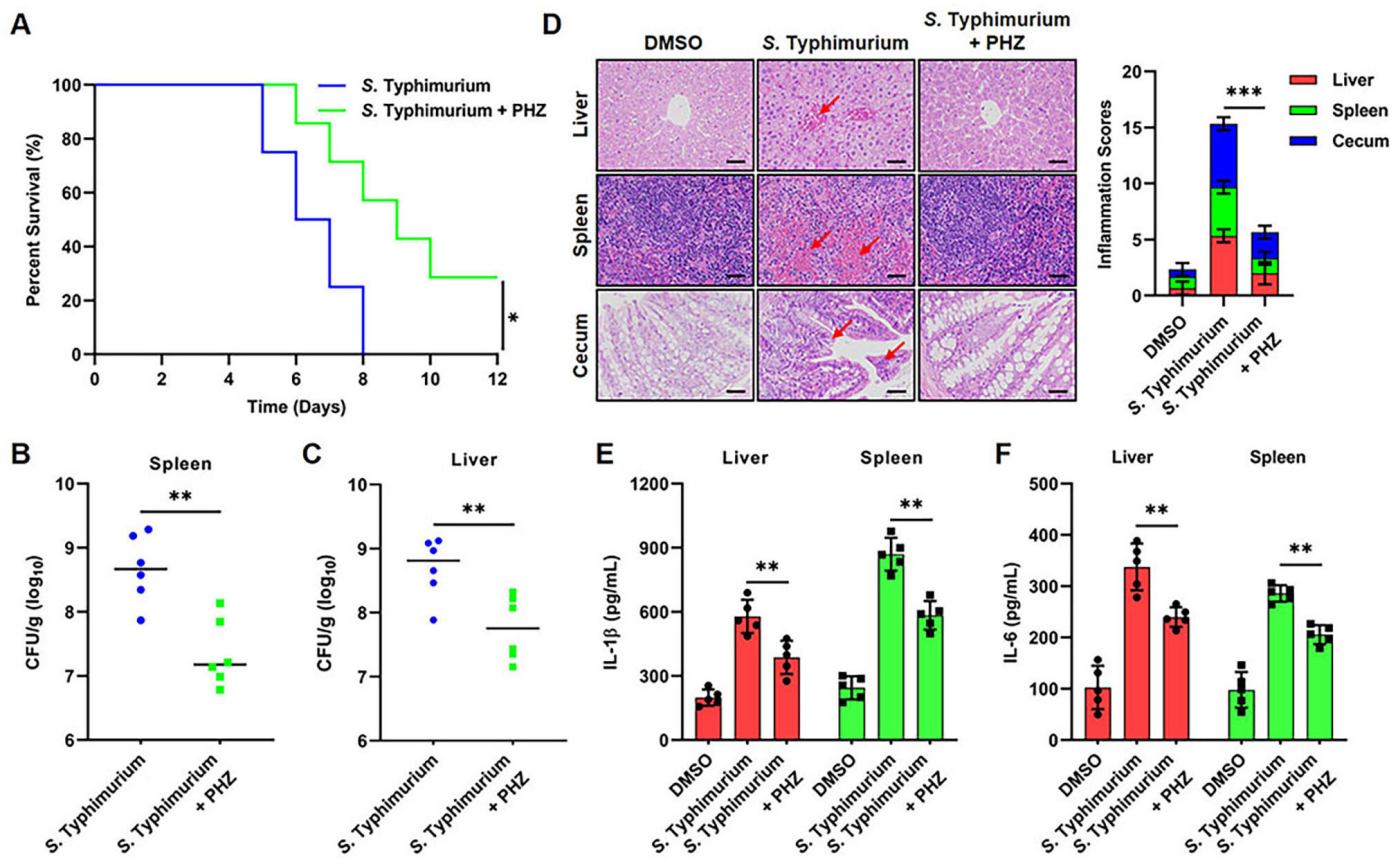
PHZ protects mice from *S.* Typhimurium infection. Mice were infected with 5 × 10^6^ CFU of *S.* Typhimurium orogastrically. **(A)** The survival rate of *S.* Typhimurium-infected mice (n= 6) untreated or treated administered PHZ. **(B, C)** Bacterial load in the livers **(B)** and spleens **(C)** of mice (n= 6) was analyzed by the CFU assay at day 4 post oral infection. The number of CFU in each organ is shown as log_10_ CFU. **(D)** Histopathology analysis of the liver, spleen, and cecum in mice collected at day 4 post oral infection (scale bar: 50 μm). **(E, F)** The levels of the proinflammatory cytokines IL-1β **(F)** and IL-6 **(G)** in the liver (red) and spleen (green) homogenates were measured by ELISA. Data presented in panel E and F are represented as the mean ± SD, n = 5. ** indicates *P* < 0.01 and *** indicates *P* < 0.001 by Student’s *t*-test **(B, C)** and one-way ANOVA **(E, F)**. Panel **(D)** are representative of three independent experiments.

Given that PHZ significantly enhances the antibacterial capacity of macrophages *in vitro*, we assessed its therapeutic efficacy in a mouse model of *S.* Typhimurium infection. Mice were orally infected with 5×10^6^ CFUs of *S.* Typhimurium, resulting in 100% mortality by the eighth day post-infection (**Figure 7A**). As an antipsychotic agent with established clinical applications, the recommended daily oral dose of PHZ for adults typically ranges from 10 to 30 mg. Based on an average adult body weight of 70 kg, this corresponds to a dosage range of 0.43-1.29 mg/kg/day. In the present study, a dose of 1 mg/kg/day was administered to mice, which is comparable to the clinically relevant dosage in humans. Compared with the *S.* Typhimurium-infected group, PHZ treatment significantly reduced mortality, indicating a protective effect of PHZ against infection-induced lethality (**Figure 7A**). To systematically evaluate the alterations in bacterial load, macroscopic lesions, and histopathological features in target organs before and after PHZ treatment, mice were orally administered 5×10^6^ CFUs of *S.* Typhimurium to establish an experimental diarrheal infection model. Compared with the infected group, PHZ significantly reduced bacterial colonization in the livers and spleens of mice (**Figures 7B,C**). Histopathological examination demonstrated a significant decrease in inflammatory cell and blood cell infiltration in both liver and spleen tissues. Additionally, intestinal sections showed reduced inflammatory cell infiltration and less damage to intestinal villi (**Figure 7D**). Consistently, The ELISA analysis demonstrated that PHZ treatment significantly reduced hepatic and splenic IL-1β and IL-6 levels in mice following *S.* Typhimurium infection (**Figures 7E,F**). Collectively, these findings indicate that PHZ confers protection against *S.* Typhimurium infection in mice.

## Discussion

**Figure 8:**
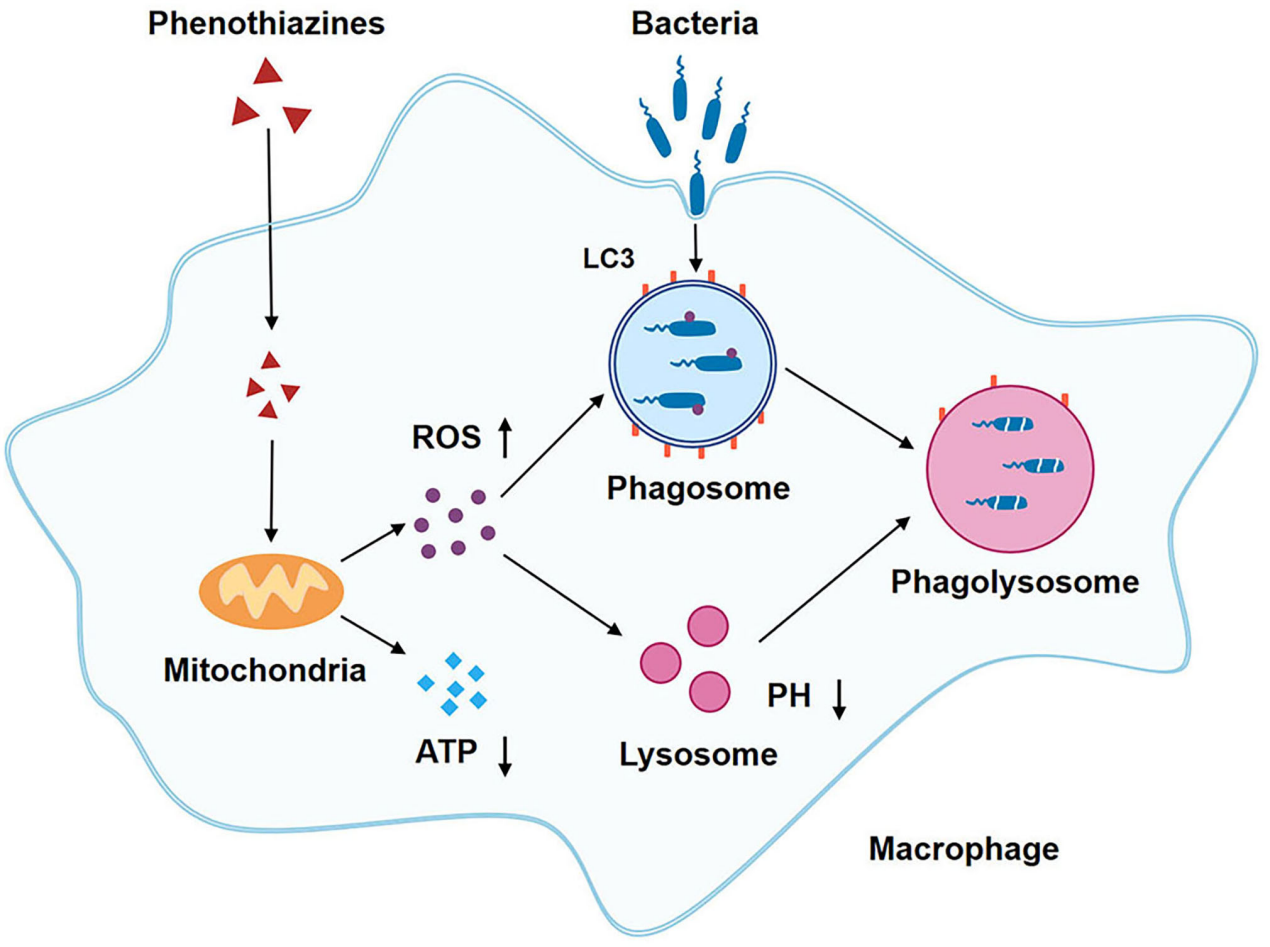
Schematic illustration of the mechanism of Phenothiazines mediated antibacterial activity in macrophage. Phenothiazines treatment induces the accumulation of ROS and a reduction of ATP levels in macrophages. The elevated ROS contributes to the clearance of intracellular bacteria through direct microbial killing, as well as by promoting lysosomal acidification and autophagy induction.

Infectious diseases have long posed a significant threat to global human health and remain a critical challenge in public health (44, 45). Emerging evidence indicates that bacterial infections are a major contributor to global health loss and have become the second leading cause of mortality worldwide, following ischemic heart disease (46). Furthermore, antibiotic resistance in bacteria is growing rapidly, making infections harder to treat and leaving more patients without effective therapeutic options (47). The development of novel antibiotics entails substantial time and financial investment (48). This creates an urgent need for alternative treatments urgently needed. HDT is a promising approach that utilizes HACs to enhance the antibacterial activities of host cells without exerting direct antibacterial effects, thereby minimizing the potential for resistance development (21, 26). Our study demonstrates that phenothiazines enhance the antibacterial activity of macrophages independently of direct antibacterial properties or adverse effects on cellular viability. These compounds clear intracellular bacteria by inducing the accumulation of ROS and promoting autophagy (**Figure 8**). Moreover, perphenazine, at clinically relevant doses, significantly reduced the burden of *S.* Typhimurium in infected mice.

Previous studies have shown that phenothiazines possess antibacterial properties and can enhance antibiotic efficacy by inhibiting efflux pumps (49–54). However, their effective concentrations are typically much higher than safe clinical levels, limiting their medical utility (55). Some phenothiazine derivatives, such as trifluoperazine, can promote the clearance of *S.* Typhimurium and *S. aureus* in macrophages at clinically safe concentration (23, 56). Our results confirm that phenothiazines suppress the replication of *S.* Typhimurium, *S. flexneri*, *S. aureus* and *L. monocytogenes* in macrophages without causing cellular damage. This supports their broad-spectrum antibacterial potential. Previous reports indicated that trifluoperazine promotes autophagy and eliminates bacteria in macrophages (57). However, we found that autophagy contributed only partially to its antibacterial effect. Macrophages are capable of eliminating intracellular bacteria through the production of ROS (41). ROS are oxygen-derived molecules characterized by high chemical reactivity and potent oxidizing capacity, generated during cellular oxygen metabolism. In macrophages, ROS contribute to host defense not only through direct antibacterial activity but also by mediating indirect antimicrobial mechanisms, including the induction of autophagy (41, 42). ROS promote the nuclear translocation of transcription factor EB (TFEB), which facilitates the transcription of lysosome-associated genes and induces the activation of autophagic pathways (58). We found that phenothiazines induce ROS accumulation. The association between ROS induction and autophagy following phenothiazines treatment may be mediated through TFEB nuclear translocation. Bacterial pathogens often trigger a metabolic shift in macrophages from oxidative phosphorylation to aerobic glycolysis upon internalization, promoting their intracellular proliferation (19, 20). Our results show that PHZ treatment significantly downregulated glycolysis-related genes, suggesting it partially reverses infection-induced metabolic reprogramming and may enhance macrophage antibacterial activity. Notably, mesoridazine differed from other phenothiazines: it neither induced ROS production nor triggered autophagy and failed to enhance macrophage antibacterial activity. Structural analysis revealed that the unique substituent at the C-2 position of mesoridazine’s parent ring distinguishes it from other compounds. This structural feature highlights a key structure-activity relationship that could guide the development of new antibacterial agents. Therefore, future studies should focus on identifying the molecular targets of phenothiazines and elucidating the mechanisms underlying ROS induction. Furthermore, the structural characteristics of their side-chain moieties should be examined in relation to ROS production, with the aim of achieving safe and precise modulation of intracellular ROS levels in macrophages.

In conclusion, our findings demonstrate that phenothiazines significantly enhance the antibacterial efficacy of macrophages through induction of ROS accumulation and autophagy. This work provides a mechanism-based lead compound for developing therapeutics against intracellular bacteria, particularly *S*. Typhimurium.

## Data Availability

The original RNA sequencing data generated and analyzed during our study have been deposited in the Sequence Read Archive (SRA) under BioProject accession number PRJNA1321154.
