# Supplementary material for: Phenothiazines enhance antibacterial activity of macrophage by inducing ROS and autophagy
Source: Front Immunol. 2025 Nov 28;16:1712724. doi: 10.3389/fimmu.2025.1712724 (PMC12698569; doi:10.3389/fimmu.2025.1712724)
Supplement: Supplementary file 1 [file DataSheet1.docx]

**Supplementary Material**


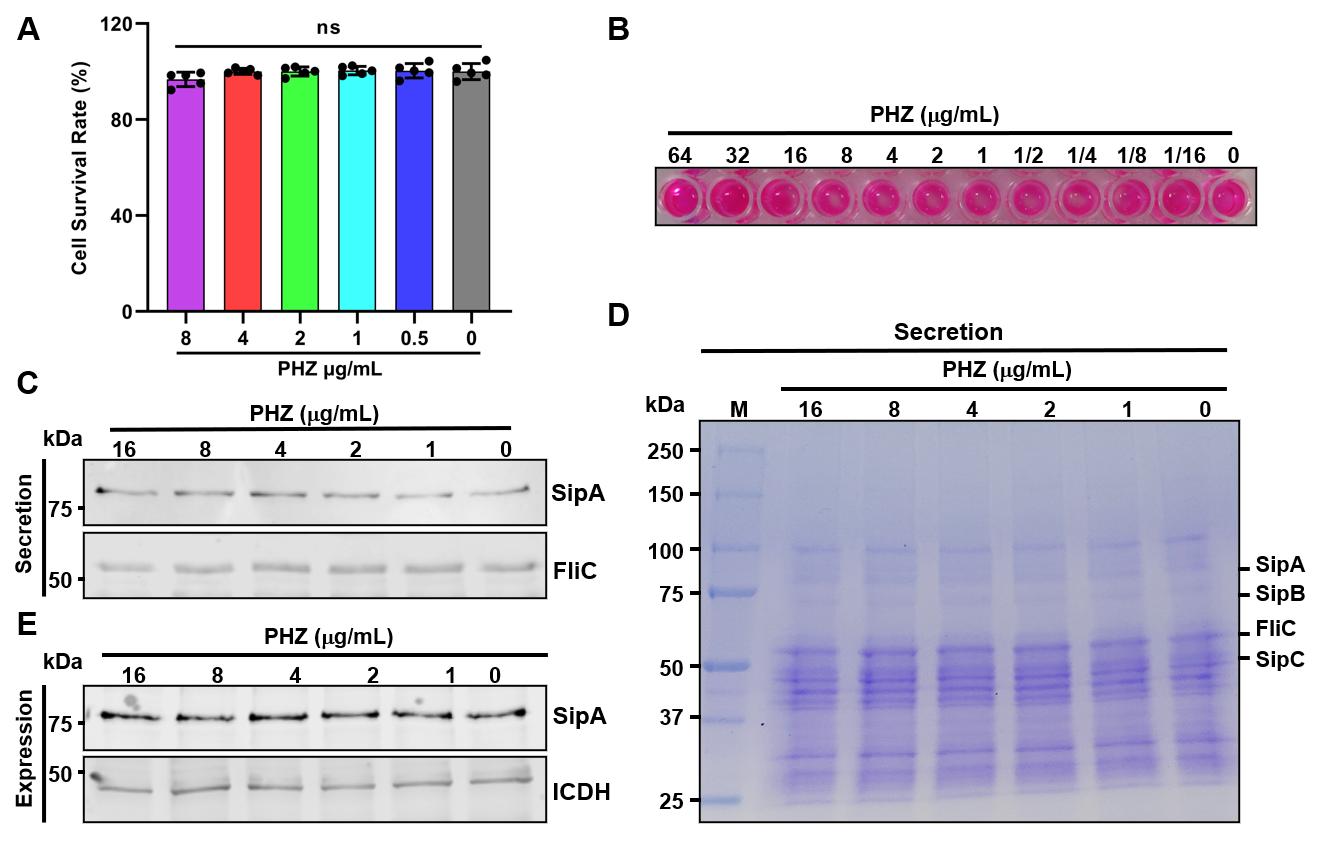


**Supplementary Figure 1 PHZ has no effect on cell viability of macrophages and growth and virulence of *S.* Typhimurium *in vitro*.**

(A) The viability of macrophages that was examined by CCK8 assay after treated with PHZ for 24 hours.

(B) The MIC of PHZ against *S.* Typhimurium was determined by the broth microdilution method.

(C-D) The secretion lever of SPI-I T3SS effectors (SipA-C) in *S.* Typhimurium growth media with or without PHZ using Coomassie brilliant blue (CBB) staining (D) and the secretion lever of SPI-I T3SS effector SipA using western blot (C). FliC (a protein secreted via the flagellar secretion system) secretion levels served as an internal reference for total secreted protein.

(E) The expression lever of SPI-I T3SS effector SipA in *S.* Typhimurium with or without PHZ using western blot. ICDH (Isocitrate dehydrogenase) expression levels were used as an internal reference for total cellular protein.

Data presented in panel A is represented as the mean ± SD, n = 5. ns indicates P > 0.05. Panel B-E are representative of five independent experiments.

**
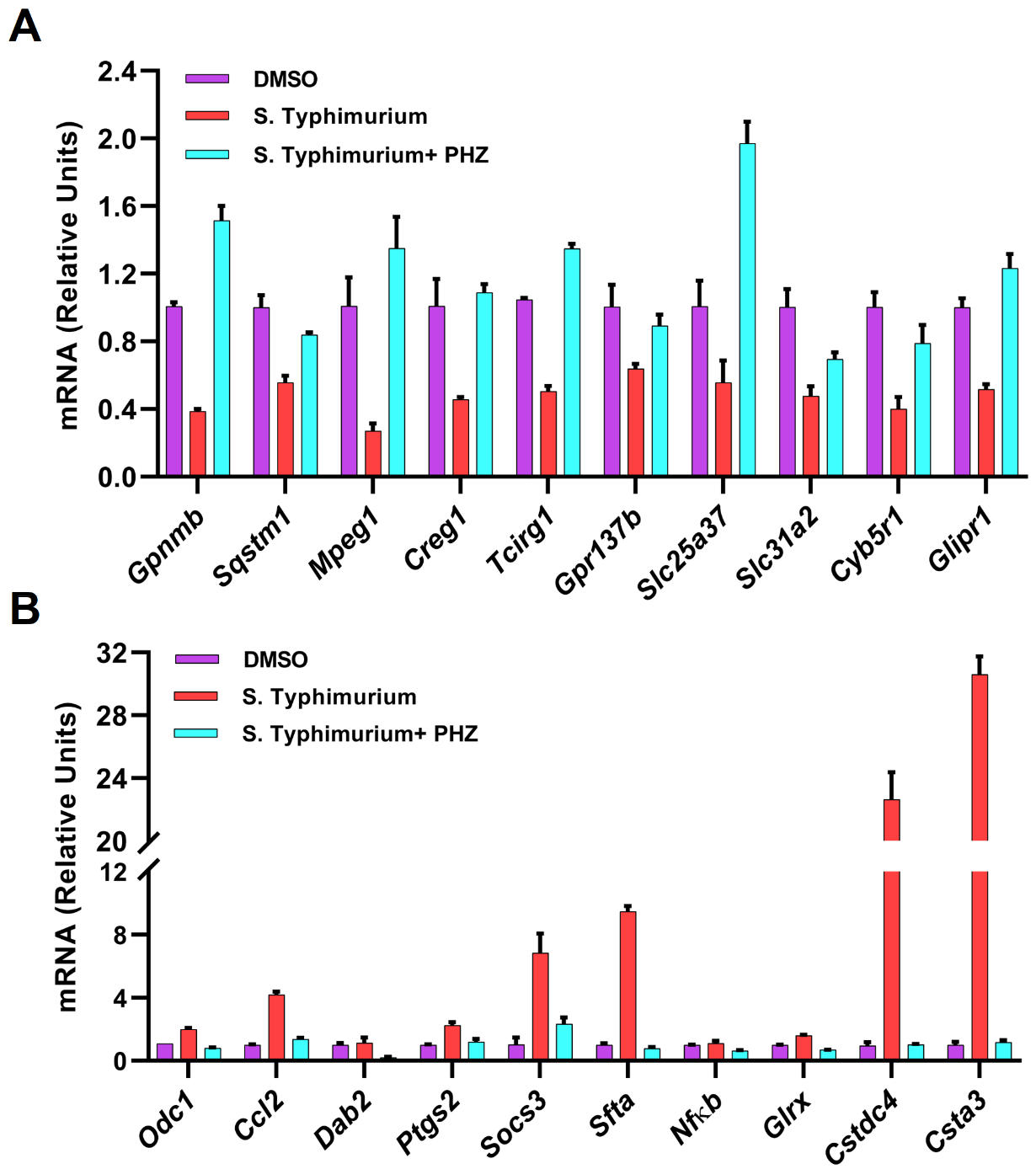
**

**Supplementary Figure 2 The relative expression levers of differentially expressed genes (DEGs).**

(A) The relative expression levels of the top 10 genes expressed in the *S.* Typhimurium+ PHZ/*S.* Typhimurium up group vs *S.* Typhimurium/DMSO down group.

(B) The relative expression levels of the top 10 genes expressed in the *S.* Typhimurium+ PHZ/*S.* Typhimurium down group vs *S.* Typhimurium/DMSO up group.

**
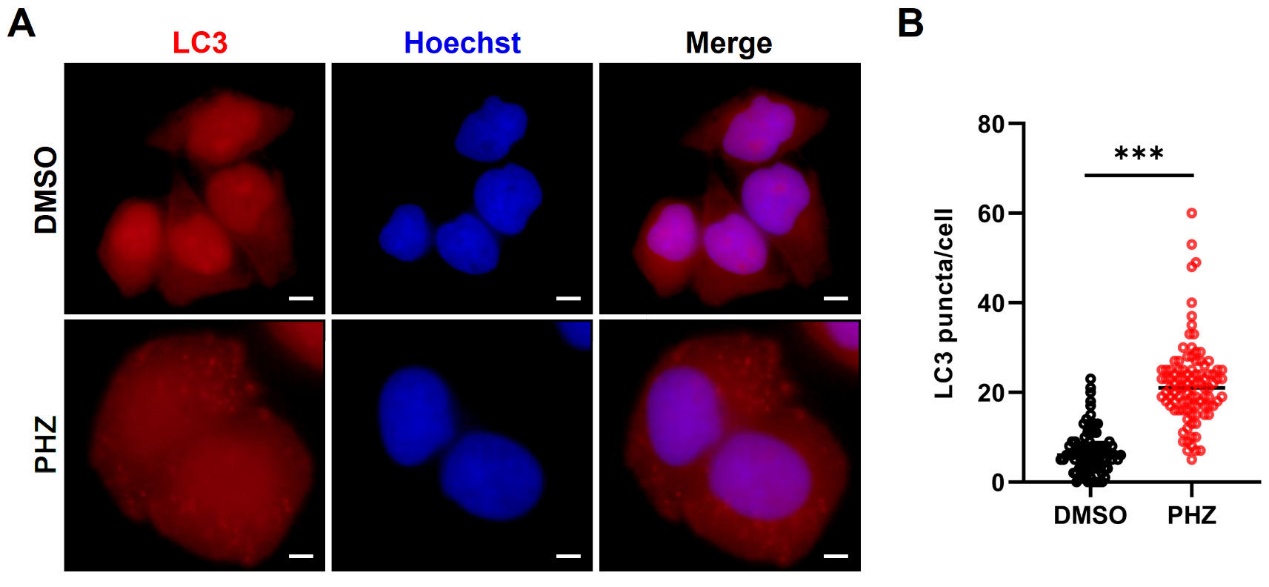
**

**Supplementary Figure 3 PHZ induces LC3 puncta in naive macrophages.**

(A) Detection by indirect immunofluorescence of LC3 (red) in naive macrophages, treated with PHZ for 8 h (scale bar: 10 μm). Hoechst (blue) was used to visualize nuclei.

(B) Determination of the number of LC3-positive puncta per cell.

Data presented in panel B is represented as the mean ± SD, n = 100. *** indicates *P* < 0.001 by Student's *t*-test. Panel A is representative of three independent experiments.

**
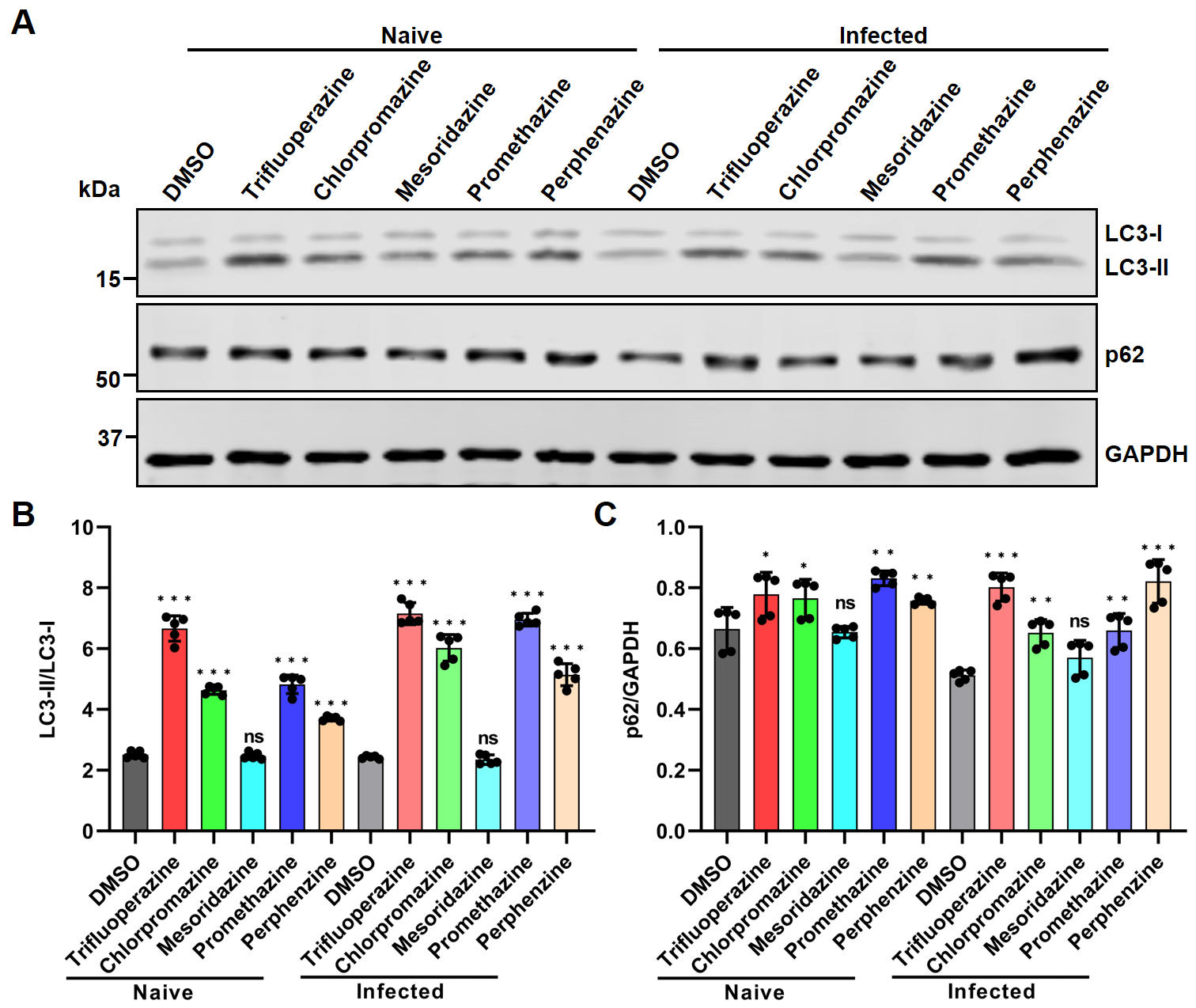
Supplementary Figure 4 Phenothiazines induce autophagy in macrophages.**

(A-C) Representative immunoblot of the expression of LC3, p62, and GAPDH in naïve and *S.* Typhimurium-infected cells treated with phenothiazines (4 μg/mL) (A). Protein quantification performed by ImageJ of LC3-II compared to LC3-I (B) and p62 compared to GAPDH (C).

Data presented in panel B and C are represented as the mean ± SD, n = 5. ns indicates *P* > 0.05, ** indicates *P* < 0.01 and *** indicates *P* < 0.001 by one-way ANOVA. Panel A is representative of five independent experiments.

**
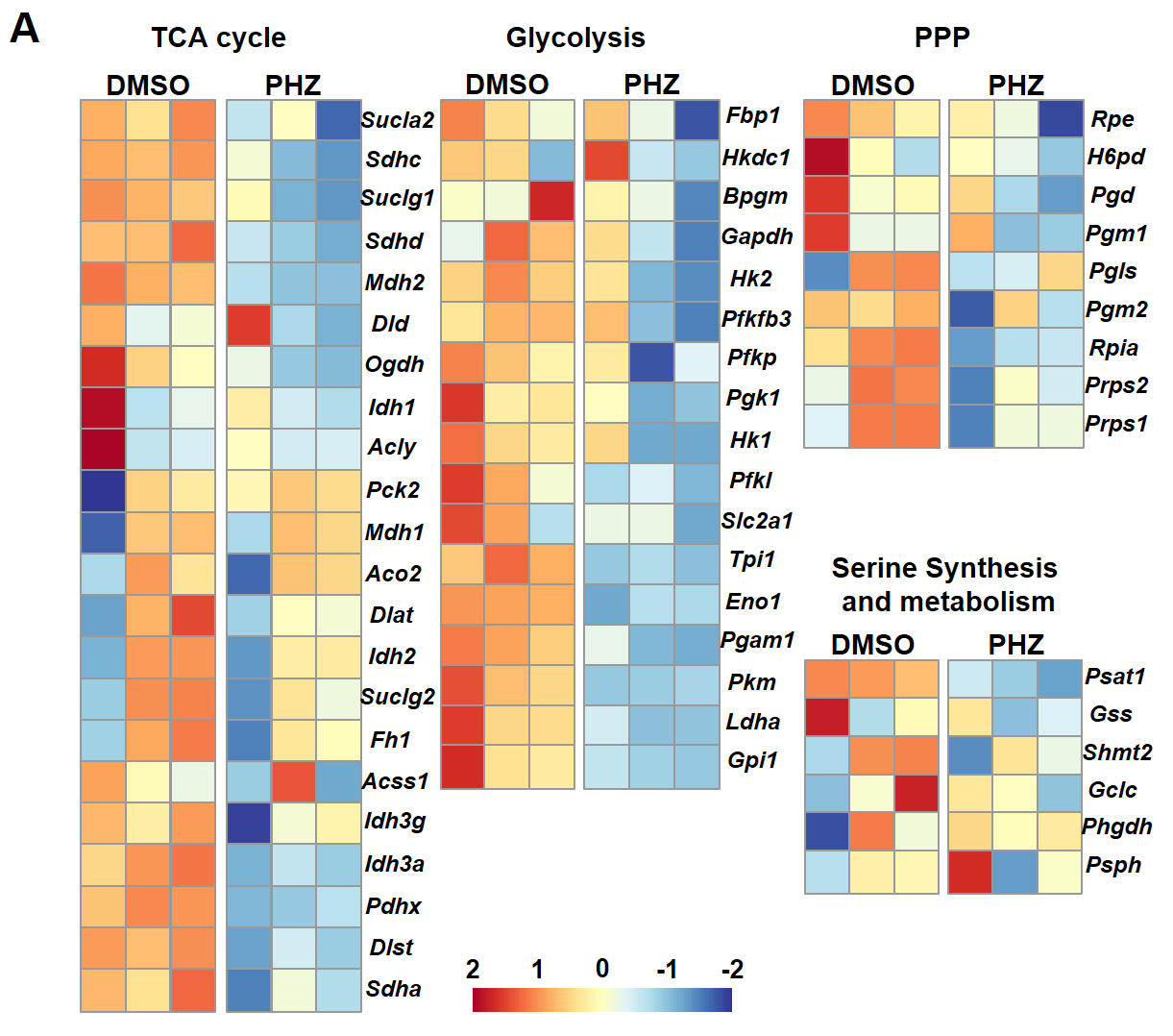
**

**Supplementary Figure 5 PHZ modulates glucose metabolism in naive macrophages.**

(A) Heatmap of the expression profiles of glucose metabolism genes in naive cells treated with PHZ. Each column corresponds to one donor. Data were normalized to determine the log ratio with respect to the median expression of each gene.

**
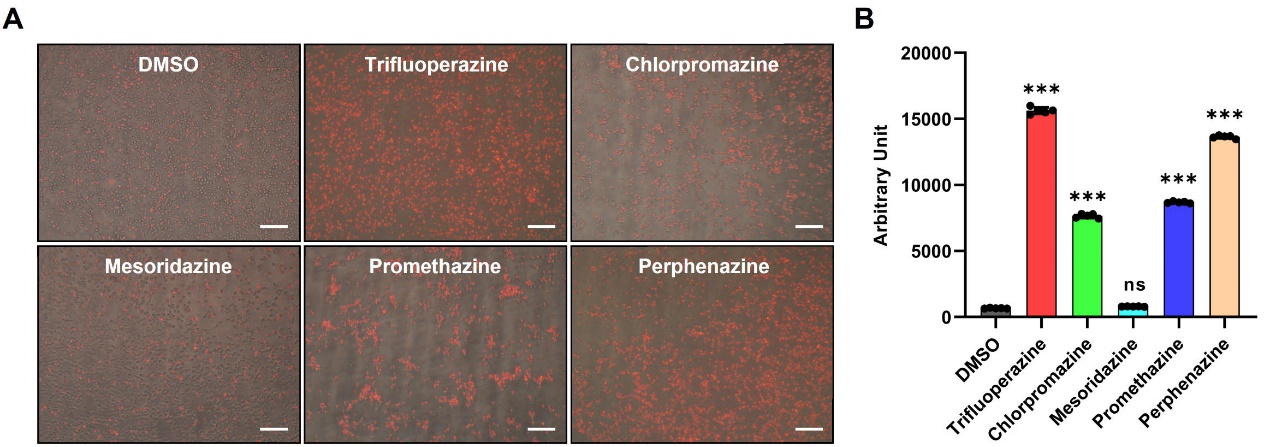
**

**Supplementary Figure 6 Phenothiazines induce the accumulation of ROS in *S.* Typhimurium-infected macrophages.**

(A-B) Raw 264.7 cells were infected with *S.* Typhimurium at MOI= 10 for 30 mins and then treated with phenothiazines (4 μg/mL) and gentamicin for an additional 8 h. After 8 h of treatment, cells were labelled with DHE (red), then fluorescence was analyzed by confocal microscopy (A) (scale bar: 50 μm). The quantification of DHE staining (B) were performed using BioTek Synergy H1.

Data presented in panel B is represented as the mean ± SD, n = 100. ns indicates *P* > 0.05, *** indicates *P* < 0.001 by one-way ANOVA. Panel A is representative of three independent experiments.
